# An Assessment of Secondary Clinical Disease, Milk Production and Quality, and the Impact on Reproduction in Holstein Heifers and Cows from a Single Large Commercial Herd Persistently Infected with Bovine Viral Diarrhea Virus Type 2

**DOI:** 10.3390/v12070760

**Published:** 2020-07-15

**Authors:** Natália Sobreira Basqueira, Jean Silva Ramos, Fabricio Dias Torres, Liria Hiromi Okuda, David John Hurley, Christopher C. L. Chase, Anny Raissa Carolini Gomes, Viviani Gomes

**Affiliations:** 1 Department of Internal Medicine, College of Veterinary Medicine and Animal Science, University of São Paulo, São Paulo 05508-270, Brazil; na_sobreira@usp.br (N.S.B.); ramos.jan@outlook.com (J.S.R.); torres@axysanalises.com.br (F.D.T.); 2 Biological Institute, 1252 Conselheiro Rodrigues Alves Ave, Vila Mariana, São Paulo 04014-900, Brazil; okuda@biologico.sp.gov.br; 3 Food Animal Health and Management Program, College of Veterinary Medicine, University of Georgia, Athens, GA 30602, USA; djhurley@uga.edu; 4 Department of Veterinary and Biomedical Sciences, South Dakota State University, Brookings, SD 57007, USA; christopher.Chase@SDSTATE.EDU; 5 Department of Veterinary Medicine, Federal University of Paraná, Curitiba 80040-380, Brazil; anny.gomes7@gmail.com

**Keywords:** diarrhea, bovine respiratory disease, milk production, somatic cells count (SCC), reproductive performance, BVDV persistent infection

## Abstract

The aim of this study was to evaluate secondary clinical disease, milk production efficiency and reproductive performance of heifers and cows persistently infected (PI) with bovine viral diarrhea virus type 2 (BVDV type 2). PI animals (*n* = 25) were identified using an antigen capture ELISA of ear notch samples. They were distributed into three age groups: ≤ 12 (*n* = 8), 13 to 24 (*n* = 6) and 25 to 34 (*n* = 11) months old. A control group of BVDV antigen ELISA negative female cattle that were age matched to the PI animals was utilized from the same herd. The PI group had a 1.29 higher odds ratio for diarrhea than controls (*p* = 0.001, IC95% = 1.032–1.623) and 1.615 greater chance of developing bovine respiratory disease (BRD) (*p* = 0.012, IC95% = 1.155–2.259). The age at first insemination (*p* = 0.012) and number of insemination attempts required to establish the first pregnancy (*p* = 0.016) were both higher for PI than controls. Milk production was higher for control cows than PI cows during most of the sampling periods. Somatic cell counts (SCC) were higher in PI cows than the controls at all sampling points across lactation (*p* ≤ 0.042). PI cattle had a higher incidence of disease, produced less milk, a higher SCC, and poorer reproductive performance than control cattle in this study.

## 1. Introduction

Bovine viral diarrhea virus (BVDV) is a ubiquitous infectious agent that affects the productivity and reproduction efficiency of both dairy and beef cattle. BVDV is responsible for significant monetary losses to producers that have been estimated to range between $0.50 to $687.80 US dollars per infected animal per year [1]. Losses have been reported to be associated with morbidity, mortality, premature voluntary culling, reduced slaughter value, stillbirths, abortion and other reproductive losses, the cost of veterinary services and treatments, the cost of replacement stock, the costs of additional labor and reduction in milk production [1,2,3].

The documented clinical impacts of BVDV infection provide the rationale for continual BVDV testing in the herd [4]. The clinical disease associated with BVDV infected cattle can present as a myriad of effects. These range from mild and unapparent infection to severe disease, leading to rapid death. The disease observed depends on the properties of the infecting strain, and the immune and physiological state of the host. Host factors such as gestational age at the time of exposure, general immunocompetence, immunotolerance to BVDV viral antigens due to prior exposure or vaccination, and the level of physical, social and environmental stress impact the severity and outcome of the disease [5].

The typical clinical signs and symptoms associated with BVDV infection have been reported in both acutely infected and persistently infected (PI) cattle [6,7]. These signs and symptom can routinely be induced during experimental challenge with any of several commonly used strains [8,9,10]. However, challenge with BVDV under experimental conditions often does not reproduce the full variety of manifestations of the disease seen in the field. Specifically, there is a lack of authoritative information about the typical clinical findings throughout the lifespan of PI cattle [11]. This is, in part, due to the short lifespan of PI cattle, which is often less than one year [12].

We have observed that there are few studies that have been published detailing the associated clinical picture of BVDV-associated secondary diseases in PI cattle. The diseases most frequently detected in PI cattle infected with the major BVDV 1 subtypes (a, b, c) were diarrhea (41%), bronchopneumonia (20%), and their combination (9%). In addition, neurological signs were observed at necropsy in 10% of the BVDV infected PI cattle. Further, abortion, laminitis and other diseases of the hoof, weakness, and anemia were only occasionally observed in PI animals [13].

Kane et al. (2015) described an outbreak of BVDV type 2a that results in abortion and PI births, despite neonatal deaths. Many of the PI cattle died as yearlings. Seventeen of 36 died with lesions consistent with mucosal disease, whereas six died without gross lesions, and two were euthanized because of chronic ill thrift. The 11 PI animals appeared healthy and were sold for slaughter. The virus population of BVDV type 2a in PI animals from this outbreak differs in size and diversity, and it could be used as a quantifiable phenotype correlated with clinical presentation of BVDV such as growth rate, congenital defects, viral shed and cytokine expression [14].

BVDV PI animals are often difficult to breed successfully. Further, herds with PI cattle can have reproductive problems. BVDV has been identified in the ovaries and uterus of PI animals [15]. The virus appears to inhibit conception through physiological alterations that induce morphologic changes in the ovaries leading to an increased interval between calving. In addition, BVDV inhibits normal embryo cleavage and reduces the number of viable embryos produced during in vitro fertilization [15,16,17]. BVDV can also induce congenital defects during fetal development, abortions (in 6% to 10% of fetuses), and stillbirth at any point in gestation [18]. Thus, PI cattle may have a significant role in poor reproductive function in herds.

A dairy herd containing PI infected animals generally has a higher incidence of mastitis (both subclinical and clinical), and frequently has a higher somatic cell count (SCC) than comparably managed BVDV free herds [19,20,21,22]. The effects of BVDV on milk production include: an average loss of 368 kg of milk, 9.35 kg of protein and 10.2 kg of fat per cow per lactation [23].

The majority of studies estimating the losses caused by BVDV infection have been reported for animals with acute infection. There is little reliable information about the impact of PI cattle with respect to secondary clinical disease, efficacy of reproduction, or its impact on milk production and quality. Thus, the aim of this study was to collect data about associated secondary clinical diseases, document reproductive efficacy, and document milk production and quality for Holstein PI heifers and cows that had naturally acquired infection with BVDV type 2 viruses.

Here, we present data collected from one large commercial herd with a well-documented history of BVDV infections over a long period. Prior to the PI screening in this herd, BVDV-associated diseases and production losses were inferred from farm records. Historically, this farm had a high incidence and recurrence of bronchopneumonia in young calves, characterized by an interstitial bronchopneumonia or fibrinosuppurative bronchopneumonia. In August 2013, the etiological cause of bovine respiratory disease (BRD) was investigated for ten young heifers. Two of these animals were positive in transtracheal wash for BVDV as detected by RT-PCR [24]. Between 2013 and 2015, BVDV was confirmed for some cases of fetal infection by the detection of serum BVDV antibody in calves before colostrum intake. In addition, neonate calves were found to be BVDV positive by RT-PCR [25,26]. In this herd, our team investigated the evidence of specific antibodies against the BVDV p80 protein in heifers from 1 to 390 days of age (*n* = 585). We detected low levels of p80 antibody in serum over the first 120 days in 10–20% of the calves. There was an abrupt rise in the number of p80 positive to 70% of the calves being serum positive after 120 days of age. This was associated with a higher mortality of calves in the post-weaning period due to anaplasmosis and babesiosis [27]. In 2015, mucosal disease was observed in a heifer during the post-weaning period. This herd also had a decline in reproductive performance. The associated reduction in the production of calves was not initially associated with BVDV infection. However, evidence linking BVDV to calf disease lead to a global screening for PI cattle in the herd in 2016.

Based on this history, the study presented here was conducted to determine the impact of naturally occurring BVDV PI animals on production parameters.

## 2. Materials and Methods

### 2.1. Herd Management

This study was performed on the largest commercial dairy farm in Brazil. It is located in São Paulo State (22°21′25″ S and 47°23′03″ W). The research was approved by the Committee for Ethics in Animal Use of the School of Veterinary Medicine and Animal Science from University of São Paulo in 1 May 2017 (Protocol number 5131190216). Written informed consent was obtained from the farm responsible.

This dairy herd was composed of approximately 3700 Holstein cattle. There were 1750 cows in lactation, with an average daily milk production of 38.5 L per cow. Calves were housed in individual suspended cages (1.0 × 0.9 m) from birth until they were abruptly weaned at about day 75 of age. They were transferred to group housing of 9, 18 or 36 heifers as they got older. The pens contained a sand bed, common drinking water trough, and a bunker with free choice calf starter diet (Guabi, São Paulo, Brazil). The calves had free access to grass (Tifton) pasture inside the pen.

Beginning at 10 months of age, heifers were kept in a confinement pasture with about 100 animals per unit. These heifers were enrolled into the standard reproductive protocols for the farm at about 13 months of age when they reached about 1.20 m tall at the withers. The majority of the heifers had entered the reproductive protocol by 13 to 14 months of age and their average weight was 380 kg. In the reproduction protocol, each heifer received a dose of prostaglandin (MSD Animal Health, Montes Claros, Minas Gerais) weekly. The heifers were inseminated when they demonstrated they were in heat. Pregnancy diagnosis using ultrasound (Mindray, Shenzhen, China) was performed every 21 days, and heifers previously inseminated, but not pregnant at this time were submitted to the fixed-time artificial insemination protocol, or fixed-time embryo transfer. A majority of the heifers (92–93%) were pregnant by 17 months of age. They were kept in a confinement pasture with about 100 animals per pasture with free choice TMR ration formulated and produced in the farm, water and access to grass pasture until the seventh month of gestation.

In the eighth month of pregnancy, heifers were moved to the maternity pen. This pen had a cross-ventilation system and held 200 heifers or dry cows. After calving, lactating cows were housed in a force cross-ventilated barn, divided into six pens, with about 290 animals in each pen.

During early calfhood, passive BVDV control was assumed to be provided by the transfer of maternal BVDV antibody in colostrum (see BVDV vaccine protocol below). Calves were fed a total volume of 5 L of fresh maternal colostrum (containing ≥50 g/L of IgG, documented using a colostrometer (MS Schippers, Campinas, Brazil), divided into two feedings. Both feedings were delivered within the first 18 h after birth.

### 2.2. Herd Vaccination History

The young heifers were vaccinated against BVDV (using a killed commercial vaccine—Cattle Master^®^ GOLD FP 5/L5, Zoetis, Parsippany-Troy Hills, NJ, USA), beginning at 60 days of age with a priming dose. This was followed by a booster dose of the same vaccine 30 days after the first vaccination. From this point forward, all animals in this herd (heifers ≥2 months of age and cows) were vaccinated biannually with the same vaccine in April and October. This herd previously used a commercial vaccine without BVDV type 2 for several decades until 2012 (Cattle Master 4 + L5^®^, Zoetis). Subsequent vaccination utilized an inactivated BVDV 1 (5960) and BVDV 2 (53637) containing vaccine with the addition of a thermosensitive BoHV-1 (Cooper) and BPIV-3 (RLB 103), a modified-live BRSV (375), and five species of killed *Leptospira* spp. This vaccine was diluted in an immune stimulating complexes (ISCOM) adjuvant (Cattle Master^®^ GOLD FP 5/L5, Zoetis).

### 2.3. PI Screening and Choice of Experimental Groups

Ear skin samples (ear notches measuring 1 cm × 0.5 cm), were obtained from the dorsal pinna margin of each young heifer or calf (*n* = 2247) using a stainless-steel ear notching clamp (type V pig, Walmur, Porto Alegre, Rio Grande do Sul). The sample obtained was stored in a sterile microtube (Eppendorf, São Paulo, Brazil), then frozen at −20 °C until it was processed. Each sample was assessed individually for BVDV using an antigen capture, Erns antigen specific ELISA test (IDEXX BVDV Ag/Serum Plus Test, IDEXX, Westbrook, ME, USA). After 30 days, animals positive at initial screening were retested using the same protocol with new biopsy samples. This was followed by testing of all live dams and grandams of the animals identified as PI among the heifers and calves. After the removal of all PI animals identified in this herd, all newborn calves were tested monthly by ear notch sampling. The duration of PI screening lasted about 13 months from September of 2015 until October of 2016.

A total of 26 PI cattle, including 19 calves and heifers, 4 dams and 3 neonates (born after removal of PI animals from the investigated herd) were found. Twenty-five of the 26 heifers and cows were included in this study. Animals were distributed by age: ≤12 months (*n* = 8), 13 to 24 months (*n* = 6) and 25 to 36 months (*n* = 11). The control group was composed of animals free of persistent BVDV (*n* = 25). They were selected as aged matched pairs for the PI cows and heifers.

### 2.4. Virus Characterization

Blood samples were collected via jugular venipuncture using an 18 gauge × 2.5 cm single sample needles into two 8 L glass tubes (Vacutainer ACD Solution A REF 364606, BD Diagnostics, San Jose, CA, USA) from all animals in the study. The blood collected in ACD solution A were processed for buffy coat isolation as previously described by Harpin et al. [28] and stored at −80 °C.

For BVDV characterization, the RNA was extracted from the buffy coat using TRIZOL LS reagent (Invitrogen, Thermo Fisher Scientific Inc., Waltham, MA, USA), according to manufacturer’s instructions and stored at −80 °C until molecular testing was performed. For RT-PCR, a one-step RT-PCR kit (Access Quick RT-PCR System, Promega Corporation, Madison, WI, USA) was utilized and a set of primers from the 5′ UTR region (5′ TAG CCA GCT CCT TAG TAG GAC 3′ and 5′ ACT CCA TGT GCC ATG TAC AGC 3′) were selected. This allowed the detection of all BVDV strains (types I, II and Hobi-like) [23]. Using a total volume of 25 µL, the reaction was performed using 12.5 µL AcessQuick Master mix 1× (Tris-HCl, KCl, dNTP, MgCl_2_ and Taq Polymerase), 1.25 µL of sense primer and 1.25 µL of anti-sense primer (1 mol/µL), 0.5 µL of reverse transcriptase enzyme AMV, 4.5 µL of nuclease-free water and 5 µL of isolated RNA. The amplification cycle was 45 °C for 45 min for disassociation and reverse transcriptase activation at 94 °C for 5 min. This was followed by 40 cycles of 94 °C for 30 s, 57 °C for 30 s and 72 °C for 30 s. An incubation at 72 °C for 5 min allowed the final extension. The assay was held at 4 °C following amplification. Amplicons were visualized on 1.5% agarose gels by horizontal electrophoresis. The gels were stained with Gel Red at a dilution of 1:150 (Biotium, Fremont, CA, USA). The products were visualized and measured using a transilluminator under ultraviolet light (302 nm). The samples were considered positive, as compared to the BVDV positive control, if the product was 29 bp. The standard 100 bp ladder (Thermo Fisher Scientific Inc. Waltham, MA, USA) was used to establish the product size.

From 19 BVDV positive samples by RT-PCR, five were submitted to sequencing to verify which type was involved in this herd. Three PIs aged from 1 to 12 months of age, 1 PI from 14 to 24 months of age, and 1 PI from 25 to 32 months of age were selected so that there was at least one PI representative of each age group. The PCR products were purified using the Wizard DNA and PCR Clean-up kit (Promega™, Madison, WI, USA) following manufacturer’s instructions. The reaction consisted of the BigDye 3.1 Xterminator kit^®^ (Applied Biosystems™, Foster City, CA, USA) and universal BVDV primers (3.2 pmol/µL) from the 5′ UTR region. The total reaction volume was 10 µL. The sequencing cycle consisted of 1 cycle of 95 °C for 1 min, 35 cycles of 95 °C for 30 s, 50 °C for 15 s and 60 °C for 4 min. This amplification was followed by holding the product at 4 °C to preserve the DNA. The sequencing reaction plate was precipitated using the BigDye Xterminator purification kit according to manufacturer’s instructions. Finally, the plate was applied to a 350 L Sequencer (Applied Biosystems™). The quality of sequences generated was analyzed using Sequence Analyzer software (Applied Biosystems™). The BVDV sequence data was edited using the BioEdit program, and after obtaining a consensus sequence fit, it was assessed using Blast and aligned with published BVDV sequences using the ClustalW program. The criteria utilized for building the phylogeny tree were based on a better than 98% identity with the core BVDV sequence. The MEGA 7.0 program was used to determine which algorithm method [29] could be applied to these samples for relatedness analysis, and a bootstrap of 1000 replicates was used to build a relational tree.

### 2.5. Data from the Farm Record

The farm records were utilized to document the reproductive performance, milk production and milk quality. Retrospective data correspond to the longevity of PI animals, since they were diagnosed as persistently infected at different ages and were immediately slaughtered after diagnosis. The digital record of each animal from the Dairy Comp Program^®^ (Valley Agricultural Software, Tulare, CA, USA) was used to generate the milk production records.

The reproduction records were collected from the farm register. The parameters collected were: age at first insemination, number of artificial insemination (AI) attempts required to achieve conception, the frequency of abortion, and the age of each heifer at the first calving. Fourteen of 16 PI heifers (12 to 36 months of age) were presented for artificial insemination. The success in reproduction (based on the parameters collected), was compared to control heifers paired for age and size (*n* = 14).

Eight PI heifers calved. These PI heifers were assessed relative to their BVDV negative pair heifer (*n* = 8). Data were also collected comparatively for the quantity and quality of the milk produced from the monthly milk report of the farm based on program records. All data collected were assessed relative to windows for days in milk: M1 = 15–39 days; M2 = 44–73 days; M3 = 74–104 days; M4 = 107–132 days; M5 = 139–166 days and M6 = 167–195 days. Not all data was recorded for each cow at each interval, because all PI cows were slaughtered immediately after being confirmed positive for BVDV by antigen ELISA results, removing them from the study in different phrases of lactation.

We were able to collect data for immediate post-partum diseases in eight PI and eleven paired cows with respect to the treatment given. The data for the treatment of cows for metritis, mammary edema, retained placenta, ketosis, clinical mastitis and bovine respiratory disease were collected.

### 2.6. Clinical Scores

Clinical scores were collected for the heifers (both PI and paired controls) at the initiation of the study. Over the course of the study, all pairs remaining at each assessment point were collected for health status. The health status of each heifer (or cow) was evaluated using a scoring system developed at the University of Wisconsin (https://www.vetmed.wisc.edu/fapm/svm-dairy-apps/calf-health-scorer-chs/). At each assessment point, a clinical examination was performed for each pair of heifers or cows, beginning on the day of the second BVD ear notch collection. The fecal score was recorded as 0 (normal consistency), 1 (pasty, semi-formed), 2 (pasty with large amount of water), or 3 (watery, fluid feces with fecal content in the perineum and on tail). The BRD Score was compiled from the rectal temperature, evidence of cough, character of nasal secretion, presence of ocular secretion, observation of abnormal head and ear positioning. These were classified on a scale from 0 to 3 for each observation, representing the intensity of each clinical manifestation. Animals having a fecal score of 2 or 3, or a summed BRD score of ≥4.0 were considered to have clinical disease.

### 2.7. Statistical Analysis

Statistical analysis was performed using the Statistical Package for the Social Science (SPSS) version 19.0 (IBM Corporation, Armonk, NY, USA).

The association between experimental groups (PI and paired control) for the prevalence of diarrhea, BRD, post-partum disease and the rate of successful calving was evaluated using either Chi-square or Fisher’s exact test. Fisher’s exact test was chosen when the groups had less than 5 pairs represented. Binary logistic regression was performed to estimate the odds ratios and confidence intervals of 95% between outcomes in the paired heifers (cows).

The normality of continuous variables was tested using the Shapiro–Wilk test. If the data were not normally distributed, the variables were transformed using one of the following: quadratic root, log10 or inverse transformation. Student’s t-test for normally distributed independent samples, and the Mann–Whitney U test for samples that were not normally distributed were performed to compare between groups for parametric and non-parametric analysis, respectively. The time-series analyses of milk production and milk composition were evaluated by using a one-way ANOVA for repeated measures coupled with a Bonferroni post-hoc test to compare data across time within each experimental group.

## 3. Results

### 3.1. Identification of PI Animals and the Outcomes for PI Heifers and Cows

PI screening was conducted on samples from 2247 heifers and calves from young animals born prior to the start of the testing program, and on the cows in the herd that had male or stillborn calves. Thirty-four of 2247 (1.5%) were BVDV infected. BVDV positive animals included: 19 aged 1 to 12; seven aged 13 to 24, and eight aged 25 to 36 months.

Results of the second round of testing indicated that 19 of the remaining 30 animals (four animals died or were euthanized before retesting) were confirmed as PI. Following this round of testing, all live dams (*n* = 11) and grandams (*n* = 4) of the PI heifers and calves were tested. Four dams (25 to 36 months of age) were found to be PI. After the removal of the PI cows from the herd, all newborn calves were tested on a monthly basis as they were born. Three additional PI calves (of 103 new calves) born over the next two months were identified with no additional PI calves identified during the next nine months on this farm.

### 3.2. Virus Characterization

**Figure 1:**
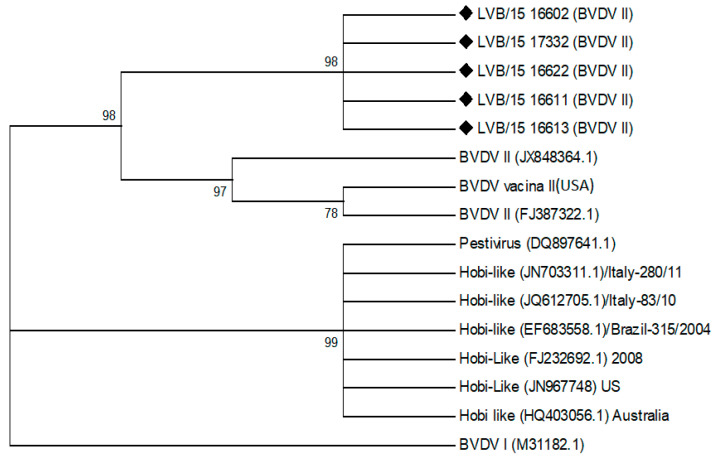
Phylogram displaying the relationships of the nucleotide sequences obtained in the current study as aligned against bovine viral diarrhea virus (BVDV) and other pestivirus sequences reported in GenBank. The phylogenetic tree was constructed using a maximum likelihood algorithm under the Kimura 2 evolutionary model with 1000 bootstrap replicates testing.

Nineteen of 25 (76%) buffy coat samples from confirmed PI cows tested were positive for BVDV. All positive samples in the RT-PCR appeared to be closely related BVDV type 2 viruses. Five of these positive samples were subjected to sequencing to confirm the genetic relatedness based on their having suitable quality for sequencing and representing the diversity of the age groups established in the study. The BVDV virus 5′ UTR sequences were analyzed and grouped in a maximum likelihood tree. The phylogenetic tree is shown in Figure 1, and it was observed that the five samples were all identified as BVDV II strains with 98% of confidence. This was based on published BVDV II strain sequences. No BVDV virus was recovered from any of the control animals using the RT-PCR test.

### 3.3. Impact of Being PI on Reproductive Performance

Reproductive performance was evaluated in sexually mature females (13 to 36 months of age). Artificial insemination of control pair heifers was initiated earlier (14.3 ± 1.19 months) than for PI heifers (15.58 ± 1.42 months) (*p* = 0.012). In addition, the number of artificial insemination attempts required to achieve the first successful pregnancy was greater for the PI heifers (mean of 4.0) than the paired control heifers (mean of 1.0) (*p* = 0.016). Only half of the 16 PI heifers calved during this study. All 16 paired control heifers produced a calf during the course of the study. There was an association observed between successful calving if a heifer was PI in the study (*p* = 0.003).

Only eight PI within a total of 14 heifers calved following artificial insemination. Failure to calf by PI heifers was generally due to abortion of her calf or being sent to slaughter before she could calve. In contrast, all paired control heifers (*n* = 14) calved following the first insemination. Control heifers were 1.75 more times more likely to deliver a calf than PI heifers (IC 95%, 1.112–2.755).

### 3.4. The Quantity of Milk Produced and Quality and Composition of Milk

A summary of the quantity of milk produced and of milk quality measurements are shown in Table 1. The difference between groups means (PI vs. control) for quantity of milk produced were: 4.71, 10.46, 11.38, 12.98, 21.23 and 16.63 L, respectively for the periods designated M1 to M6. Higher quantities of milk production were observed in control cows relative to PI cows during the sampling periods M2 (*p* = 0.019), M4 (*p* = 0.013), M5 (*p* = 0.002) and M6 (*p* = 0.004).

The SCC was higher in PI than paired control cows at each timepoint analyzed during the course of lactation. The differential in the quantity of milk produced between the PI and paired control cows increased over the course of lactation (*p* ≤ 0.001). In general, PI milk was more concentrated than milk from the paired controls. PI had higher fat content (in %) at the M5 sampling (*p* = 0.054), and showed a tendency for a higher percent of protein at the samplings M2 (*p* = 0.082), M3 (*p* = 0.094) and M4 (*p* = 0.078). The level of lactose was not different between the PI and paired control cows at any sampling point. No differences were observed within either of the experimental groups over the course of the experiment (M1–M6).

### 3.5. The Assessment of Clinical Signs and Disease

There was an association between the prevalence of diarrhea and the incidence of BRD that was different between the PI and control groups (Table 2). An analysis of the dataset, without respect to age, demonstrated an association between a higher diarrhea score (*p* = 0.012) and higher BRD score (*p* = 0.001) linked to the PI group of animals. When age was added as a factor in analysis, a tendency was detected (*p* = 0.074) toward a higher frequency of diarrhea in PI (33%) than control (0%) cattle of 25 to 36 months of age. There was no difference observed in diarrhea frequency among calves <12 months of age (*p* = 0.500), or heifers 12 to 24 months of age (*p* = 0.455) between groups. The PI heifers had a 1.29 greater odds ratio for development of diarrhea (fecal score ≥ 2.0) than the control heifers (*p* = 0.001, IC 95% = 1.032–1.623). This was assessed using binary logistic regression of the global PI population, without respect to age.

The frequency of animals with BRD (sum of scores ≥ 4) was higher for PI heifers (38.5%) than the paired control heifers (0%) based on analysis of the global data (*p* = 0.001). Similar results were observed for cows 25 to 36 months of age and young heifers (<12 month). For these cows, PI animals had a higher frequency of BRD (50.0%) than control cows (0%). No difference in BRD was observed for heifers 12 to 24 months of age (*p* = 0.251) between experimental groups in this study. The PI heifers had a 1.615 greater chance of developing BRD (sum of scores > 4.0) than the control heifers (*p* = 0.012, IC 95% = 1.155–2.259) when assessed using binary logistic regression analysis applied to the global population.

The relative occurrence of mammary edema (*p* = 0.322), retained placenta (*p* = 0.183), ketosis (*p* = 0.421), clinical mastitis (*p* = 0.297) and BRD (*p* = 0.297) in PI cows was determined by assessing values from eight lactating PI cows and 11 control cows (originally paired with PI heifers). We observe a higher frequency of the occurrence of metritis in PI (37.5%, 3/8) than the control cows (0%, 0/11) (*p* = 0.058). The PI cows had a 1.6 greater chance of developing metritis than the control cows (IC 95% = 0.935–2.737).

When all types of post-partum disease were combined, the total occurrence was higher in the PI cows (100%, 8/8) than in control cows (36.4%, 4/11) (*p* = 0.007). The PI heifers had a three-times greater chance of developing post-partum disease than paired control heifers (IC 95% = 1.348–6.678).

## 4. Discussion

This research was focused on understanding the impact of persistent infection with BVDV type 2 on secondary clinical disease, the quantity of milk produced and its quality, and on factors affecting reproductive efficacy in Holstein heifers and cows. The study comprehensively tested the herd and discovered a significant number of PI heifers, calves and even established cows (25–36 months of age). To our knowledge, this paper represents the first study to examine the impacts of BVDV type 2 persistent infection relevant to dairy cattle production and reproduction in a large dairy herd. Previously, Kane et al. (2015) reported information about clinical presentation in PI caused by a single strain of BVDV2, however it was in a Angus and Angus-cross beef herd.

This study collected all of the data from PI cattle with naturally acquired infections. Previous papers in the literature have almost exclusively presented the effects of induced, or naturally acquired, acute infection, or induced PI resulting from BVDV challenge. The experimental challenge cannot induce disease that has identical complexity as naturally acquired infections. It is likely that challenge models do not fully replicate the interaction between virus, host and the environment that occur in a true production setting.

The investigated herd was not closed. Animals from southern Brazil were added to the herd continually. To accelerate the genetic improvement in the herd, heifers and cows from the farm were sent to an embryo harvesting center in Paraná state in the south of Brazil. The embryos resulting from their utilization of this reproductive technology were implanted in cows from the farm of origin. The embryo transfer facility also had cows and heifers from other farms that did not utilize formal BVDV control programs. BVDV testing and vaccination for these animals from other farms was not implemented until the cows and heifers arrived at the embryo facility. After delivery of the calves, half of the new heifers resulting from this program were returned to the farm where the cows originated. Among the PI cows in this study, at least one animal infected with BVDV type 2 came from this embryo harvesting facility.

During the last two decades (1998–2018), over 300 bovine pestiviruses have been partially or fully sequenced in Brazil. These include viruses identified in a number of geographical regions, representing different backgrounds of the cattle, and from cattle presenting with diverse clinical pictures. Phylogenetic analysis of these viruses demonstrated a predominance of BVDV 1 (54.4%) in Brazil. The BVDV type 1 subgenotypes identified were: 1a (33.9%) and 1b (16.3%) most frequently, and subgenotypes-1d, -1e, and -1i at very low frequencies. The overall BVDV type 2 frequency was 25.7%, but it varied considerably by the region of the country. It reached as high as 48% of the BVDV identified in one state. HobiPeV accounted for 19.9% of the viruses identified. HobiPeV had the highest frequency in northeast Brazil [30].

Understanding regional genetic diversity of ruminant pestiviruses is important for establishing appropriate vaccine protocols. It makes sense that vaccines should include viral genotypes that are present in each region [31]. In Brazil, most commercial vaccines against BVDV contain only BVDV type 1 strains. This is in the face of epidemiological evidence of a broad distribution of the BVDV type 2 viruses among several Brazilian states. The farm where this research was conducted utilized a vaccine containing BVDV type 1a for several decades prior to our study. The vaccine protocol was changed in 2012. This was due to the introduction of the first commercial BVDV vaccine containing both BVDV type 1 and type 2 genotypes of virus.

The detection of 17 persistently infected cattle of 13 to 36 months of age was a surprise. Taylor et al. reported that only 4/51 PI animals survived longer than one year in a beef herd that was studied.

Noncytopathic (NCP) BVDV establish lifelong PI in fetal calves following infection between 40 and 120 days of gestation, prior to functional immune system development. Cytopathic BVDV strains arise from NCP strain via mutation in the NS23 gene. Superinfection of PI animals with a closely related CP virus will generate mucosal disease [32]. Darweesh et al. reported a mortality rate of 23/41 (56%) in PI cattle also infected with BVDV type 2a that subsequently developed mucosal disease. The adult PI reported in this research did not develop fatal mucosal disease [33].

The prevalence of diarrhea disease and BRD in PI cattle infected with BVDV type 2 was established by scoring fecal and respiratory signs into disease scores in this study. PI cattle had a higher prevalence of both diarrhea (22.7%) and BRD (38.1%) than the paired control animals (0%). This finding was most clear in adult animals (25–36 months of age). It is our contention that while these animals did not develop mucosal disease they did develop subacute chronic intestinal and/or pulmonary inflammatory problems that resulted in the development of secondary disease.

Bachofen et al. published a retrospective and prospective study that analyzed 86 clinical reports of PI animals (median: 12 months of age) between January 1995 to 2005. The animals were under the care of the ruminant clinic at the Department of Farm Animals, University of Zurich. Within this population, 26% (about 30 cattle) were 24 months of age, and four animals were more than three years old. Most of the cases had a history of recurrent, or untreated, diarrhea (41%), pneumonia (20%) or both together (9%). These finding are similar to the findings reported from our study.

Unfortunately, the short time between the completion of the second PI testing cycle and the PI slaughter by the farm did not allow us to conduct a complete physical examination of all PI animals. Therefore, our clinical findings were limited to an examination of the intestinal and respiratory tract in the slaughtered cattle. However, our physical examination of lactating cows allowed us to detect enlarged lymph nodes and enlarged hemolymphatic nodules throughout the body under the skin. We were also able to observe any asymmetry of the mammary gland (quarters), and identify vulvovaginitis, periodontitis and circular alopecia lesions around the eyeball. Further, all lactating PI cows had at least one post-partum disease. These included hypocalcemia, mammary edema, mastitis, retained placenta, metritis, ketosis or BRD. It is our position that this is the first report of the spectrum of post-partum disease that occurs within PI cows.

BRD is frequently reported in cattle when acutely or persistently infected with BVDV. The nasopharynx and respiratory tract are the main routes of entry for BVDV virus. BVDV antigen has routinely been detected in both the upper and lower respiratory tract of animals with BRD [34,35]. Moreover, the genetic material of BVDV has been previously detected using trans-tracheal lavage from both slaughterhouse and necropsied animals, some showing no symptoms [36,37].

This assessment of reproductive records indicated that PI heifers were older than their paired controls at their first artificial insemination attempt. This was due to a delay in the development of sexual maturity in the PI heifers. The earliest inseminations were done at 12 to 13 months of age. The heifers weighed about 350 kg and were about 1.20 m tall at the withers. Heifers should have attained at least 40% to 50% of their adult body weight (~300 kg) by the time of the first service. Stokstad and Loken reported that the PI calves born to heifers that were challenged with an experimental infection during pregnancy were found to be smaller, less active and to grow more slowly than calves from uninfected heifer dams [38].

The number of inseminations required to achieve the first pregnancy was greater for PI heifers than the paired control heifers. BVDV has been localized in ovarian tissue for prolong periods of the following acute infection with cytophatic and noncytophatic strains [18,39]. Altamiranda et al. identified NCP BVDV throughout the ovarian tissue of PI cattle. This virus appeared to be associated with alterations in the structure of the follicular regions of the ovaries. These alterations are believed to directly impact embryo development, leading to reduced rates of ovum cleavage and impaired embryo development.

Grooms, Ward and Brock evaluated the morphological differences in ovaries of six PI cows, compared to six cows with no documented BVDV, using classical histological methods. PI cows appeared to have ovarian hypoplasia and significant morphological changes in the number of tertiary, graafian, atretic, corpus luteum hemorrhagic and albicans body follicles.

Fray et al. suggested that BVDV compromises ovarian function through three mechanisms: (1) BVDV may affect the gonadotrophic function of the pituitary; (2) BVDV suppresses the plasma estrogen level affecting ovulation and estrus; (3) BVDV induces generalized leukopenia, affecting the leukocyte population of the ovaries and impairing follicular dynamics [40].

The reproductive impact in PI cows continues after calving. PI cows have reduced milk production volume and produce more concentrated milk. It is important to note that the decrease in milk production and the increase in SCC are gradual processes that persist throughout the entire lactation period. We found no previously published data about individual PI cows with respect to milk production or quality. However, the reduced milk production, high SCC, and higher occurrence of subclinical and clinical mastitis have been reported in herds with very high BVDV antibody titers. This was observed both before and immediately after the institution of significant BVDV eradication and control programs in Europe [19,20,21,22,23].

To summarize, in a single large dairy herd, we identified 25 PI cattle infected with BVDV type 2. The impact of the virus on production measures (both reproduction and milk production) appears to be significant and consistent. It appears that PI heifers are only half as successful in achieving a first calf as paired controls, and that milk production and quality are significantly poorer in PI cows. Further, PI cattle had diarrhea disease at a higher frequency and in older cows than the paired controls, and PI cattle had a greater frequency of BRD.

## 5. Conclusions

PI cattle suffered more diarrhea and BRD than their paired controls. The PI heifers had a delayed initial breeding success, and only half of PI heifers delivered a calf following their initial series of AI service. In addition, the PI cows had reduced milk production and poorer milk quality, with an increased SCC throughout lactation.

## Figures and Tables

**Table 1:** Mean and standard deviation (±SD) of the quantity of milk produced and the composition of the milk produced by PI and paired control cows (NI) over the course of lactation. SCC: somatic cell count.

Variables	Groups	M1 (15–39 DIM)		M2 (44–73 DIM)		M3 (74–104 DIM)		M4 (107–132 DIM)		M5 (139–166 DIM)		M6 (167–195 DIM)		*p*-Value
		Mean ± SD	*N*	Mean ± SD	*N*	Mean ± SD	*N*	Mean ± SD	*N*	Mean ± SD	*N*	Mean ± SD	*N*	
Yield (Liters)	PI	26.29 ± 11.77	7	24.29 ± 10.61	7	27.5 ± 5.28	6	28.4 ± 4.56	5	23.4 ± 12.82	5	26 ± 2.16	4	0.217
	Nl	31 ± 13.06	7	34.75 ± 14.71	8	38.88 ± 15.42	8	41.38 ± 8.57	8	44.63 ± 7.29	8	42.63 ± 7.82	8	0.948
	*p*-value	0.492	-	0.143	-	0.112	-	0.010	-	0.003	-	0.002	-	-
SCC (×10^5^)	PI	203.43 ± 246.21	7	308.86 ± 380.88	7	530 ± 476.28	6	1046.2 ± 970.37	5	919.2 ± 979.85	5	1045.25 ± 1156.22	4	0.555
	NI	50.57 ± 50.21	7	23.88 ± 16.99	8	41.38 ± 72.17	8	372.38 ± 978.72	8	21.75 ± 18.61	8	24 ± 14.4	8	0.445
	*p*-value	0.042	-	0.001	-	0.001	-	0.004	-	0.001	-	0.006	-	-
Lactose (%)	PI	4.66 ± 0.53	7	4.86 ± 0.3	7	4.75 ± 0.48	6	4.6 ± 0.49	5	4.72 ± 0.37	5	4.7 ± 0.44	4	0.954
	NI	4.63 ± 0.51	7	4.84 ± 0.16	8	4.79 ± 0.22	8	4.85 ± 0.18	8	4.79 ± 0.2	8	4.84 ± 0.2	8	0.941
	*p*-value	0.920	-	0.438	-	0.734	-	0.380	-	0.904	-	0.901	-	-
Fat (%)	PI	3.4 ± 0.81	7	3.77 ± 0.8	7	3.3 ± 0.36	6	3.34 ± 0.63	5	3.86 ± 0.46	5	3.38 ± 0.69	4	0.673
	NI	3.11 ± 0.38	7	3.45 ± 0.46	8	3.49 ± 0.57	8	3.18 ± 0.52	8	3.38 ± 0.27	8	3.23 ± 0.49	8	0.574
	*p*-value	0.475	-	0.712	-	0.512	-	0.710	-	0.054	-	0.961	-	-
Protein (%)	PI	2.91 ± 0.21a	7	3.07 ± 0.18ab	7	3.13 ± 0.16ab	6	3.22 ± 0.13ab	5	3.24 ± 0.05ab	5	3.35 ± 0.13b	4	0.001
	Control	2.83 ± 0.26a	7	2.88 ± 0.21a	8	2.96 ± 0.18ab	8	3.06 ± 0.16ab	8	3.16 ± 0.16b	8	3.26 ± 0.21b	8	0.003
	*p*-value	0.586	-	0.082	-	0.094	-	0.078	-	0.403	-	0.175	-	-

*N* = sample size. Difference between group means was assessed using Student’s *t* test, and changes over time using a one-way ANOVA. *p*-value was considered significant if *p* < 0.05, and tendency declared when *p* > 0.05–1.00.

**Table 2:** The percentage (number of cases/number of subjects *100) of diarrhea and bovine respiratory disease (BRD) in persistently infected (PI) and control Holstein heifers and cows.

Clinical Scores	Groups	Results	Global Population	Age (Months)		
				<12	13–24	25–36
Diarrhea (Score ≥2.0)	PI	Positive	22.7 (5/22)	12.5 (1/8)	20.0 (1/5)	33.3 (3/9)
		Negative	77.3 (17/22)	87.5 (7/8)	80.0 (4/5)	66.7 (6/9)
	Control	Positive	0 (0/25)	0	0	0
		Negative	100 (25/25)	100 (8/8)	100 (6/6)	100 (11/11)
	*p*-value		0.012	0.500	0.455	0.074
BRD (Sum of Score ≥4.0)	PI	Positive	38.1 (8/21)	37.5 (3/8)	20.0 (1/5)	50 (4/8)
		Negative	61.9 (13/21)	62.5 (5/8)	80.0 (4/5)	50 (4/8)
	Control	Positive	0 (0/25)	0 (0/8)	0 (0/6)	0 (0/11)
		Negative	100 (25/25)	100 (8/8)	100 (6/6)	100 (11/11)
	*p*-value		0.001	0.055	0.251	0.008

Global population means all PI and non-infected BVDV, without respect to age. Difference between groups was considered significant if *p* ≤ 0.05, and a tendency declared if *p* > 0.05 ≤ 0.10, using the Chi-square or Fisher exact test as necessary.
